# Changes of Serum Homocysteine and Vitamin B12, but Not Folate Are Correlated With Obsessive-Compulsive Disorder: A Systematic Review and Meta-Analysis of Case-Control Studies

**DOI:** 10.3389/fpsyt.2022.754165

**Published:** 2022-05-09

**Authors:** Sirui Yan, Hailong Liu, Yaqiong Yu, Nashu Han, Wenzhi Du

**Affiliations:** Department of Psychiatry, Mental Health Institute of Inner Mongolia Autonomous Region, Hohhot, China

**Keywords:** obsessive-compulsive disorder, folic acid, homocysteine, vitamin B12, meta-analysis

## Abstract

**Background:**

Obsessive–compulsive disorder (OCD) a complex neuropsychiatric disorder, is characterized by irresistible obsessive thinking and compulsive behavior. Folate is a member of water-soluble vitamins in the human body and sustains many normal daily activities (e.g., exercise, sleep, and memory). Homocysteine, a sulfur-containing non-essential amino acid, has been investigated in numerous psychiatric disorders (e.g., OCD). Vitamin B12 is a type of complex organic compound with cobalt contained. Moreover, vitamin B12 and folate deficiency and high levels of homocysteine were found to have an effect on brain functions and also lead to non-specific psychiatric symptoms.

**Objectives:**

This study aimed to confirm the epidemiological evidence of OCD and investigate whether vitamin B12, folate, and homocysteine have an effect on the etiology of OCD.

**Methods:**

A systematic search was conducted on eight databases (i.e., PubMed, Embase, Web of Science, the Cochrane Library, China Biology Medicine disc, China National Knowledge Infrastructure, Wanfang Database, China Science and Technology Journal Database), and the retrieval time was up to March 2021. The available articles involving patients with OCD with/without abnormal serum levels of vitamin B12, folate, and homocysteine were comprehensively reviewed and analyzed.

**Results:**

A total of 5 studies involving 309 patients were included in this meta-analysis, including 172 cases in the experimental group and 137 in the control group. The content of folate in the OCD group was not significantly different from that in the control group (SMD = −0.089, 95%CI −0.755 to 0.577, *p* = 0.794). And serum homocysteine was significantly higher in the patients with OCD (SMD = 1.132, 95%CI 0.486 to 1.778, *p* = 0.001). Vitamin B12 was significantly lower in patients with OCD (SMD = −0.583, 95%CI −0.938 to −0.229, *p* = 0.001).

**Conclusions:**

This meta-analysis shows serum high levels of homocysteine, low levels of vitamin B12, and normal folate level are closely correlated with OCD. However, high-quality case-control studies should be further conducted to explore the correlation between serum levels of vitamin B12, folate, homocysteine, and OCD.

**Systematic Review Registration:**

https://www.crd.york.ac.uk/prospero/display_record.php?ID=CRD42021262161; PROSPERO (Number CRD#42021262161).

## Introduction

According to the latest revision of diagnostic criteria for OCD^1^ in the Diagnostic and Statistical Manual of Mental Disorders (DSM-5) (1), compulsions can be observable behaviors. The prevalence rate of OCD in the populations ranges from 0.88 to 4% (2), and regional differences lead to a change in prevalence rate to a certain extent. The mean age at onset of OCD was 20.9 to 25.4 years, and the mean lifetime duration of the disease was 11.8 years. OCD is equally common in men and women, whereas it is more common in young people, divorced or separated people, and unemployed people (3). OCD is characterized by intrusive thoughts or images (obsessions), increased anxiety, and repetitive or ritualistic behaviors (compulsions) (4). According to some researches, there are abnormalities of Cortico-Striatal-Thalamic-Cortical dysfunction and impaired inhibition in patients with OCD (5).

A series of articles primarily demonstrated that a subset of the different forms of OCD is familial and thus might arise from genetic factors (6–8). A meta-analysis (9) including 37 groups of samples with 24,161 pairs of twin from 14 articles examined the heritability of obsessive-compulsive behaviors. Findings from this meta-analysis supported the hypothesis that genetic factor is critical for manifesting obsessive-compulsive behaviors. In addition, some researchers approved that OCD was because of the abnormal expression and function of some genes in the serotonergic and dopaminergic pathways (10). The “serotonergic hypothesis” (11), which proposed that OCD was primarily a disorder of the serotonin system, has received scientific support. The role of dopaminergic mechanisms in the manifestation of OCD has been confirmed (12–15). Furthermore, it is progressively confirmed that glutamate and GABA^2^ also have an effect on the occurrence of OCD (16–18). In addition, the caudate nucleus has been involved in the pathophysiologic process of OCD, and lots of studies reported hyperactivity of the bilateral caudate nucleus bilaterally in both the adult and pediatric patients (19–21). However, despite all these cognitive causes, the etiology of OCD is not completely clear.

Amounts of evidence support the cognitive behavioral therapy and selective serotonin reuptake inhibitors (SSRIs) have been as the treatment of OCD (22–26).

As revealed, homocysteine is correlated with both current depression and a history of depression, and a higher concentration of homocysteine might increase the risk of depression (27). Amounts of evidences show that folate and vitamin B12 are important nutrient elements involved in the physiological and pathological functions of the neuropsychiatric system (28–30). The incidence of neuropsychiatric findings in patients with vitamin B12 deficiency has been reported to be 4–50% (31). More recently, folate and vitamin B12 have been reported in a large amount of literature as treatments or supplements for the treatment of psychiatry disease (32–36). The interaction among folate, vitamin B12, and homocysteine, genetic factors, and metabolism of neurotransmitters have significance in patients with OCD (37–39). As the research on folate and its carbon unit metabolism, homocysteine and other psychiatric disorders has been progressed, researchers have been attempting to determine the correlation between them with OCD, whereas inconsistent results were achieved (37–40). Thus, this meta-analysis was conducted to investigate the epidemiological evidence of serum levels of folate, homocysteine, and vitamin B12 in OCD.

## Materials and Methods

### Literature Search Strategy

This meta-analysis was based on the statement of the Preferred Report Item for Systematic Reviews and Meta-analysis (PRISMA) (41). A systematic search was conducted on eight databases (i.e., PubMed, Embase, Web of Science, the Cochrane Library, CBM, CNKI, WANFANG DATA, China Science and Technology Journal Database). The search strategy included the search terms: (1) “OCD” or “obsessive-compulsive” or “Obsessive-Compulsive Neuroses” or “Anankastic Personality”; (2) “Vitamin M” or “Vitamin B9” or “Pteroylglutamic Acid” or “Folic Acid, Monopotassium Salt” or “Folic Acid,” (DL)-Isomer” or “Folvite” or “Folacin” or “Folate”; (3) “Homocysteine” or “2-amino-4-mercaptobutyric acid” or “L-Isomer Homocysteine”; (4) “Vitamin B12” or “Cyanocobalamin” or “Cobalamin” or “Eritron.” Date search was limited from inception to 1 March 2021. All the abstracts were evaluated in accordance with the inclusion and exclusion criteria to verify the suitability for meta-analysis, and articles were added by screening references of included articles conditionally (Table 1).

**Table 1:** Literature search strategy.

**Number**	**#1**	**#2**	**#3**	**#4**
Mesh term	Obsessive-compulsive disorder	Vitamin B12	Folic acid	Homocysteine
Other term	Obsessive-compulsive disorder	B 12, Vitamin	Vitamin M	2-amino-4-mercaptobutyric acid
	Obsessive-compulsive	Vitamin B12	Vitamin B9	2 amino 4 mercaptobutyric acid
	Disorder, obsessive-compulsive	B12, Vitamin	B9, Vitamin	Homocysteine, L-Isomer
	Disorders, obsessive-compulsive	Cyanocobalamin	Pteroylglutamic acid	Homocysteine, L Isomer
	Obsessive-compulsive disorder	Cobalamins	Folic acid, monopotassium salt	L-Isomer Homocysteine
	Obsessive-compulsive disorders	Cobalamin	Folic acid, monosodium salt	
	Neurosis, obsessive-compulsive	Eritron	Folic acid, potassium salt	
	Neuroses, obsessive-compulsive		Folic acid, (DL)-isomer	
	Neurosis, obsessive-compulsive		Folvite	
	Obsessive-compulsive neuroses		Folacin	
	Obsessive-compulsive neurosis		Folate	
	Anankastic personality		Folic acid, (D)-isomer	
	Anankastic personalities		Folic acid, calcium salt (1:1)	
	Personalities, anankastic		Folic acid, sodium salt	
	Personality, anankastic			

*Search strategy: #1 AND #2, #1 AND #3, #1 AND #4*.

### Article Selection and Eligibility Criteria

Original articles matching the following eligibility criteria were included: (1) cross-sectional, case-control, or cohort study design; (2) involving a sample of patients with clinically diagnosed OCD, with/without a comparison group (at least 10 people in each group); (3) reported vitamin B12, folate, and homocysteine as outcomes and diagnosed with the Structured Clinical Interview for DSM-4/DSM-5; (4) provided sufficient raw data for calculation; (5) had clear inclusion and exclusion criteria (intellectual disability, growth disorders, psychotic disorders, substance and alcohol abuse, history of endocrine disorders and pregnant or breastfeeding patients or more than 60 years old, taking supplements or drugs in recent 1 year, not have a history of major mood disorder, dementia, intellectual disability, or psychosis in their first-degree relatives) for participants; (6) published in English or Chinese. The exclusion criteria included: (1) reviews, letters, case reports, or conference abstracts; (2) duplicated or overlapping data. All sifting process was conducted by two researchers (i.e., SiRui Yan and Hailong Liu) independently. Their respective evaluation results were compared, and any discrepancies were resolved by discussion.

### Data Extraction and Quality Assessment

In total, three independent researchers (i.e., SiRui Yan, Hailong Liu and Yaqiong Yu) extracted the information from eligible articles. Baseline data extracted from the respective manuscripts and supplements are presented later: the first name of the author, year of publication, country, ethnicity, study design, sample size, mean age, gender ratio, comparison group, diagnostic scales, and also diagnostic criteria. To accurately evaluate the methodological quality of eligible articles, three researchers (i.e., SiRui Yan and Yaqiong Yu and Nashu Han) employed the Newcastle-Ottawa Scale (NOS) (42) for the evaluation. Article quality was evaluated on the aspects of the participant selection, comparability, outcome ascertainment, and data processing. Scale score ≥7 was identified as high quality, moderate quality = 4–7, while a score below 4 was classified as poor quality.

### Statistical Analysis

The odds ratio (ORs) for dichotomous data was estimated, and standard mean differences (SMDs) for continuous data with the corresponding 95% CIs were also estimated. The chi-squared test and I^2^ statistic were performed to identify heterogeneity among articles. Under insignificant heterogeneity (I^2^ <50% or *p* > 0.1), a fixed-effect model was used to estimate the pooled effect size; otherwise, the random-effect model was used. To determine potential sources of heterogeneity between articles, sensitivity analysis, and meta-regression were conducted using prespecified variables (e.g., study design, ethnicity of the participants, outcome measurement tool). The Egger's test and Begg's test were performed to detect publication bias. Furthermore, all the data synthesis and analysis were conducted using Stata version 11.0 (StataCorp, College Station, TX, USA).

## Results

### Literature Search and Study Selection

**Figure 1:**
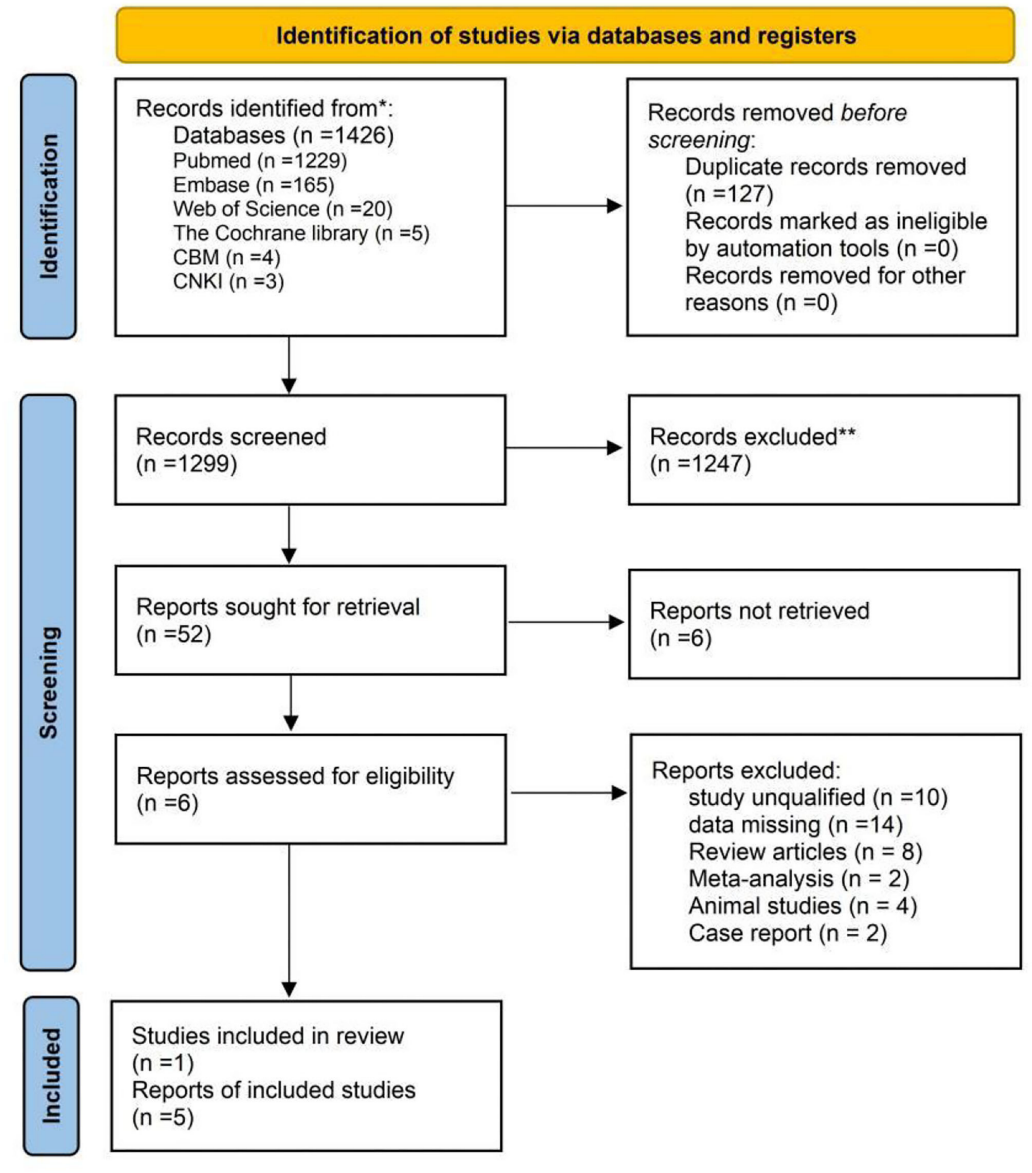
PRISMA 2020 flow diagram for new systematic reviews which included searches of databases and registers only. *Consider, if feasible to do so, reporting the number of records identified from each database or register searched (rather than total number across all databases/registers). **If automation tools were used, indicate how many records were excluded by a human and how many were excluded by automation tools.

A total of 1,426 records were found in the initial search strategy (PubMed 1229, Embase 165, Web of Science 20, the Cochrane Library 5, CBM 4, CNKI 3, WanFang Data 0, Technology Journal Database 0), and 127 duplicates were removed. A total of 1,299 records were excluded for irrelevant themes or unsuitable types of articles after the titles and the abstracts were screened. In accordance with the eligibility criteria, 40 articles were excluded through a careful review of the full texts. In summary, 5 articles involving 172 cases of OCD were selected for the meta-analysis (Figure 1).

### Characteristics of Included Studies

The last remaining 5 articles (37–40, 43) were included in this study. Table 2 lists an overview of the characteristics for 5 eligible studies, with 1 study only reporting the number of participants, and all of them were published in English. All the studies were case-control published from 1988 to 2021, 3 were conducted in Turkey (37–39), one in Iran (40) and one in Israel (43). All the cases of patients with OCD were recruited by the clinical hospitals. All of the participants were Caucasian. Sample sizes of participants ranged from 23 to 52 people, 309 in total, and men and women were represented similarly (172:137). Most of the mean age of patients with OCD ranged from 12.4 to 44.5 years. The control groups in the 5 articles were all healthy people. A total of 309, 185, and 110 participants were included about folate (172 in OCD group and 137 in control group), homocysteine (110 in OCD group and 75 in control group), and vitamin B12 (149 in OCD group and 114 in control group), respectively. Samples in all 5 studies were venous blood after overnight, and 2 studies reported the methods used to analyze the samples (37, 38), while 3 did not (39, 40, 43). Fasting Scores of the NOS or the AHRQ-11 in most articles (*n* = 4) reached 6 or higher (37–40), suggesting an overall good/moderate quality of the included articles (Table 3).

**Table 2:** Characteristics of included studies.

**References**	**Ethnicity**	**Country**	**N (D, ND)**	**Gender (% Male) (D, ND)**	**Age (D, ND)**	**Sampling time**	**Measure of determination**	**Obsessive-Compulsive Diagnosis (Scales)**	**Diagnosis Criteria**
Atmaca et al. (37)	Caucasian	Turkey	23/23	34.8/43.5	29.1 ± 6.3/28.4 ± 7.5	Sample venous blood at 07:00 −08:00 a.m. after overnight fasting	Folate: E170 hormone autoanalyzer	Yale-Brown Obsessive-Compulsive Scale (Y-BOCS)	DSM-4
							Homocysteine: Homocysteine kit (ELISA)		
Esnafoǧlu et al. (2017)	Caucasian	Turkey	52/30	50/46.7	14.7 ± 2.3/14.2 ± 2.6	Sample venous blood after overnight fasting	Folate: chemiluminescent micro particle Folate binding protein ARCHI-TECT Folate assay with commercial kits	Yale-Brown Obsessive-Compulsive Scale (Y-BOCS), KOVAKS depression scale, State-Trait Anxiety Inventory (STAI-I, STAI-II)	DSM-5
							Vitamin B12: chemiluminescent micro particle Intrinsic Factor ARCHI-TECT B12 assay with commercial kits		
							Homocysteine: chemiluminescent immunassay method using kits		
Eslamzadeh et al. (40)	Caucasian	Iran	32/32	31.3/34.4	37.50 ± 11.48/36.78 ± 10.66	Sample venous blood after overnight fasting	N	Yale-Brown Obsessive-Compulsive Scale (Y-BOCS)	DSM-5
Türksoy et al. (39)	Caucasian	Turkey	35/22	11.4/13.6	34.0 ± 10.5/33.1 ± 8.3	Sample venous blood after overnight fasting	N	Hamilton Depression Rating Scale (HAM-D), Hamilton Anxiety Rating Scale (HAM-A), Yale-Brown Obsessive-Compulsive Scale (Y-BOCS)	DSM-4
Hermesh et al. (43)	Caucasian	Israel	30/30	N	N	sample venous blood at 8:30-9:00 a.m. after at least 8 h fasting	N	N	N

*Values reflect mean ± SD in each group.

**Table 3:** Quality assessment of included studies.

**References**	**Study population selection**	**Intergroup comparability**	**Measurement of exposure factors**	**Jadad Score**
Atmaca et al. (37)	3	2	2	7
Esnafoǧlu and Yaman (38)	2	2	2	6
Hermesh et al. (43)	1	2	2	5
Eslamzadeh et al. (40)	3	2	2	7
Türksoy (39)	3	2	2	7

### Assessment Tools for Obsessive–Compulsive Disorder

Three clinical diagnostic criteria, i.e., DSM-5 (The Diagnostic and Statistical Manual of Intellectual Disability, 5th ed), DSM-4, and DSM-4-TR, were employed separately in included articles to evaluate anxiety disorder. On the whole, 7 different validated screening scales were used for evaluating obsessive symptoms. The Yale-Brown Obsessive-Compulsive Scale (Y-BOCS) is the most common tool. Furthermore, there was a problem that thresholds adopted to screening scales might differ from each other in different articles when defining anxiety symptoms.

### The Correlation Between Obsessive-Compulsive Disorder and Serum Levels of Folate

**Figure 2:**
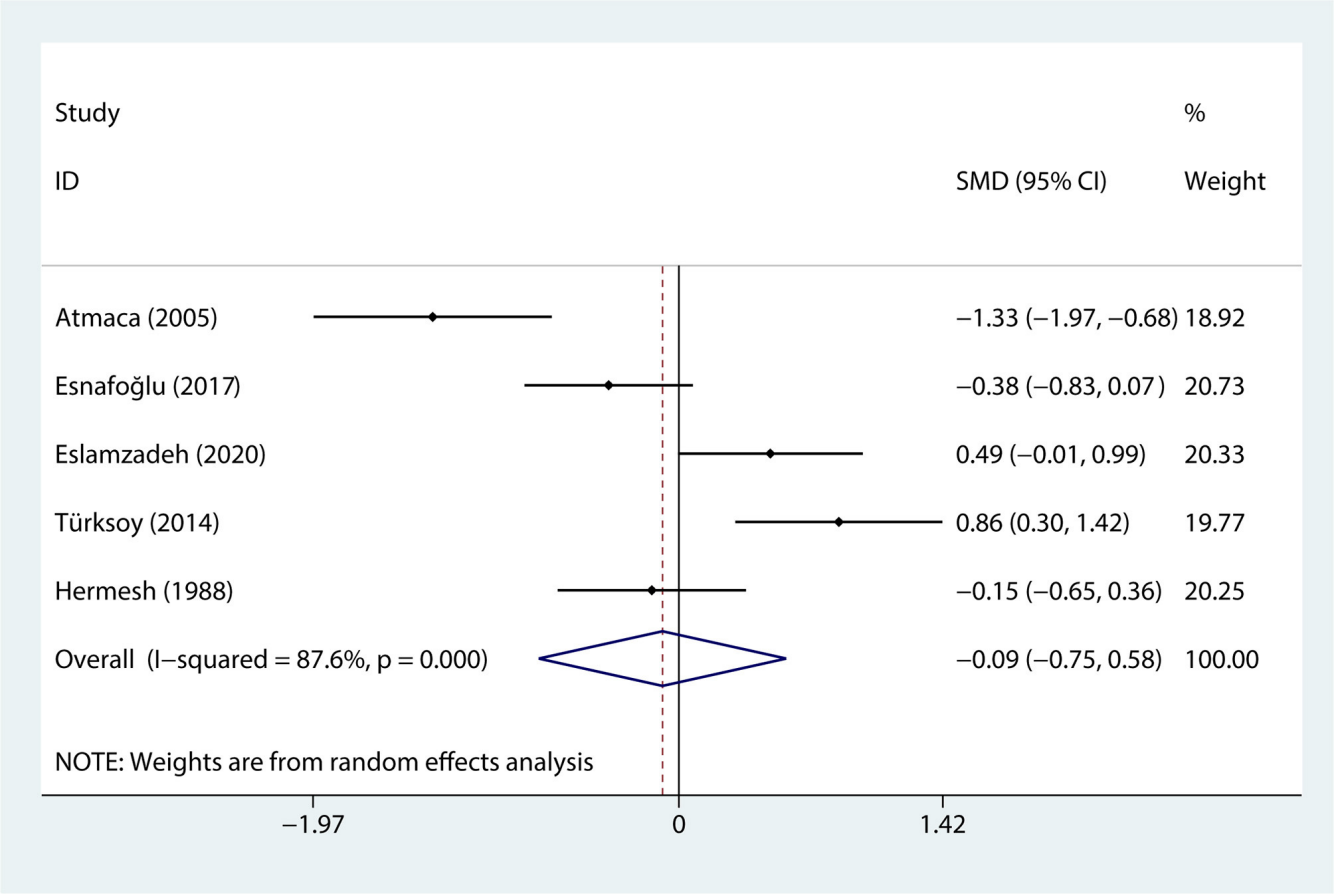
The correlation between obsessive-compulsive disorder and folate.

Data regarding serum levels of folate were provided in 4 studies with a total of 309 patients were analyzed (37–40, 43). The content of serum levels of folate in the OCD group was not significantly different from that in the control group (SMD = −0.089, 95% CI −0.755 to 0.577, *p* = 0.794, Figure 2) with obvious heterogeneity (*p* = 0.000, I^2^ = 87.6%, Figure 2).

### The Correlation Between Obsessive–Compulsive Disorder and Serum Levels of Homocysteine

**Figure 3:**
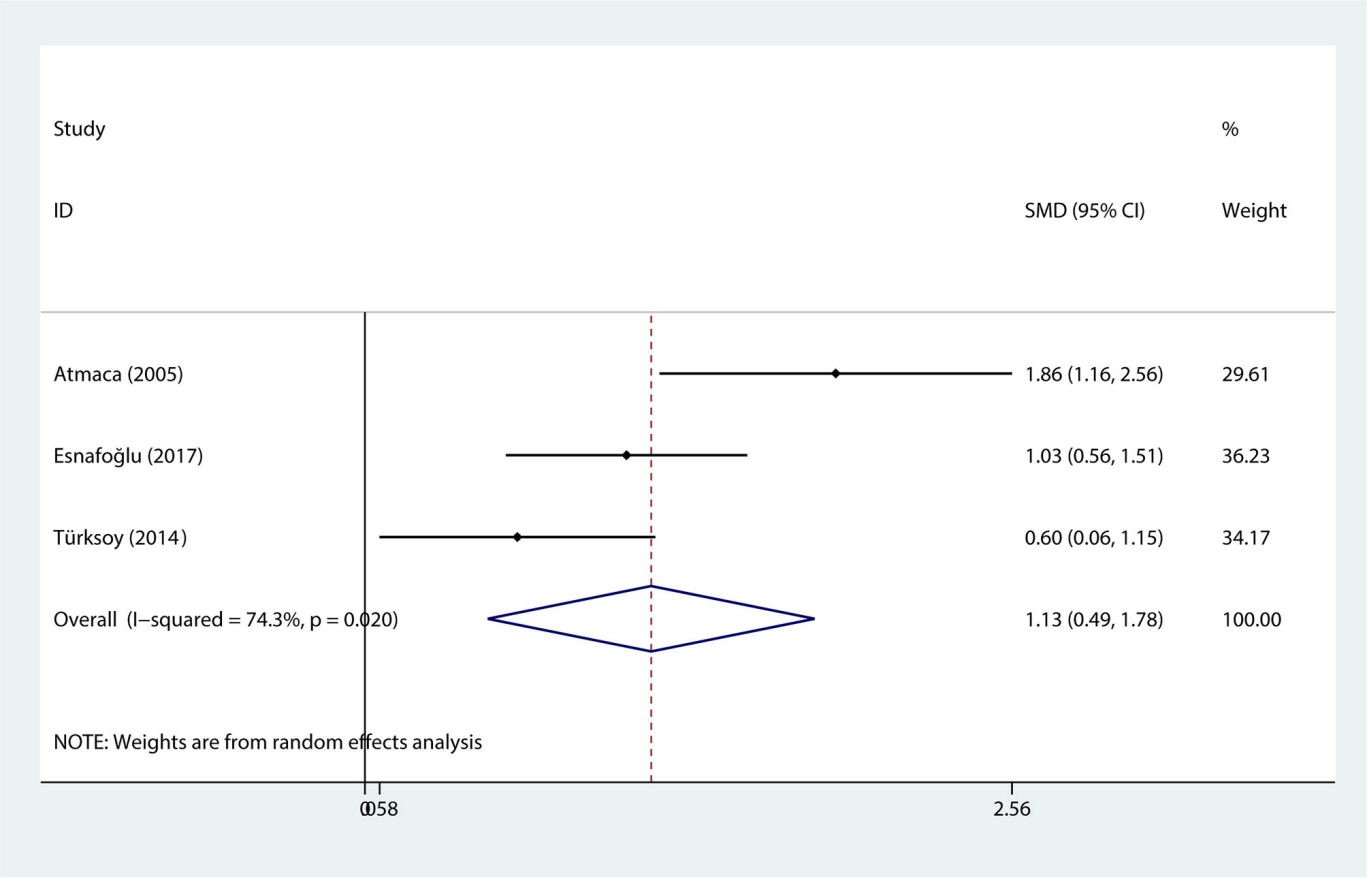
The correlation between obsessive-compulsive disorder and homocysteine.

Data regarding serum levels of homocysteine provided in 3 studies with a total of 185 patients were analyzed (37–39). The content of serum levels of homocysteine in the OCD group was not significantly different from that in the control group (SMD = 1.132, 95% CI 0.486 to 1.778, *p* = 0.001, Figure 3) with significant heterogeneity (*p* = 0.020, I^2^ = 74.3%, Figure 3).

### The Correlation Between Obsessive–Compulsive Disorder and Serum Levels of Vitamin B12

**Figure 4:**
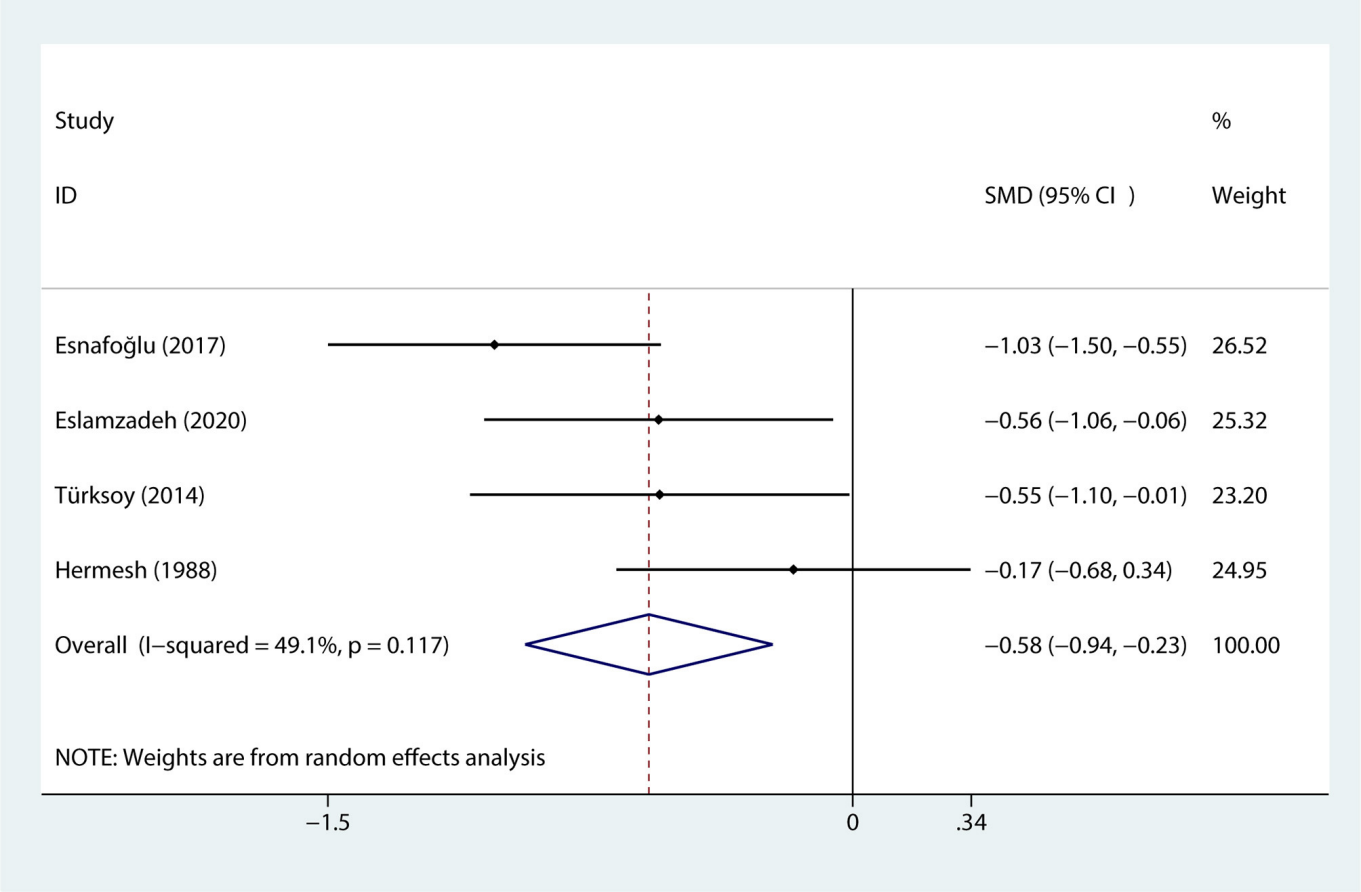
The correlation between obsessive-compulsive disorder and vitamin B12.

Data regarding serum levels of vitamin B12 provided in 4 studies with a total of 110 patients were analyzed (38–40, 43). The content of serum levels of vitamin B12 in the OCD group was significantly lower than that in the control group (SMD = −0.583, 95% CI−0.938 to−0.229, *p* = 0.001, Figure 4) and there was no obvious heterogeneity (*p* = 0.117, I^2^ = 49.1%, Figure 4).

### Sensitivity Analysis

**Figure 5:**
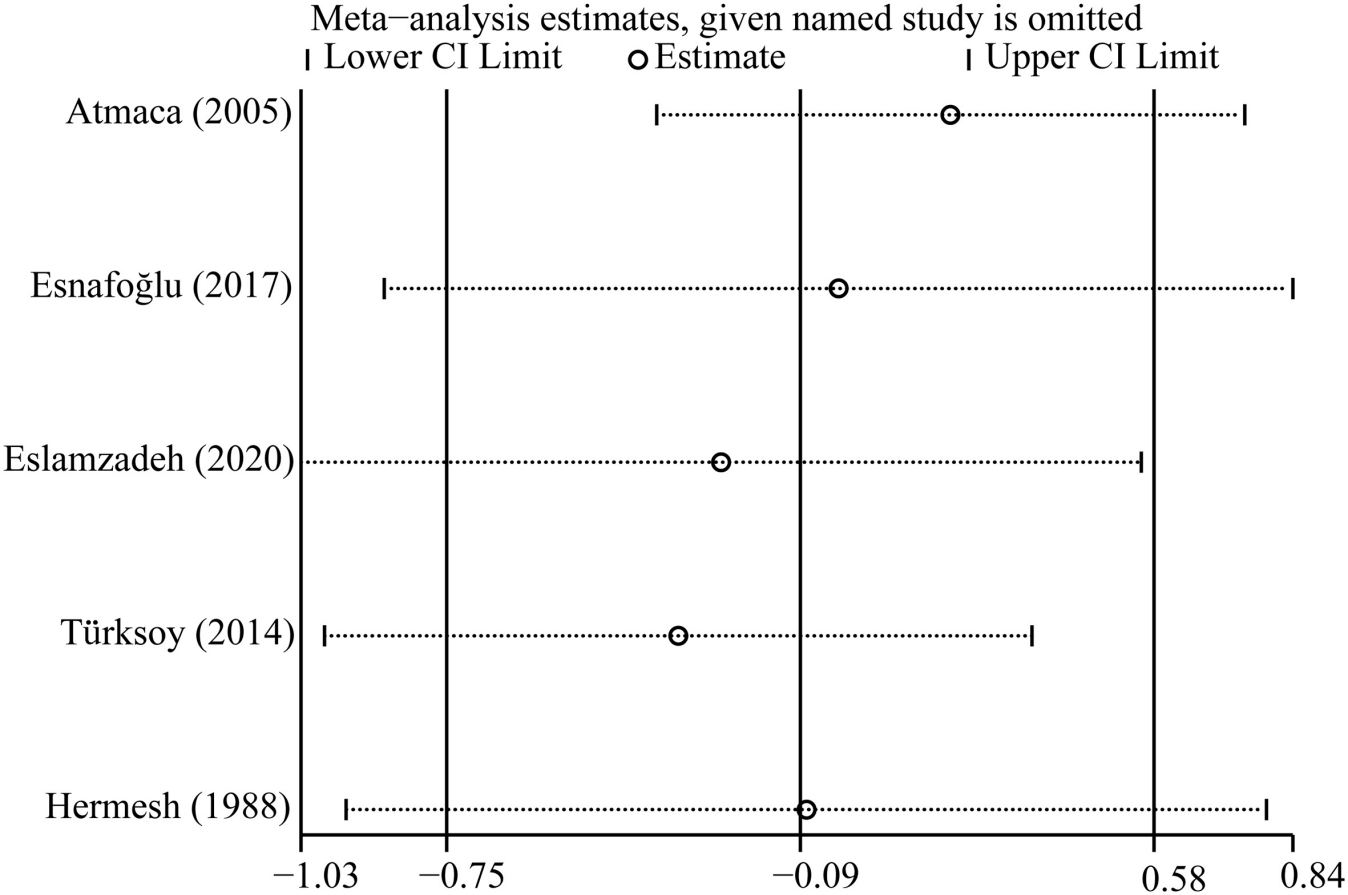
Sensitivity analysis of included studies on folate.

**Figure 6:**
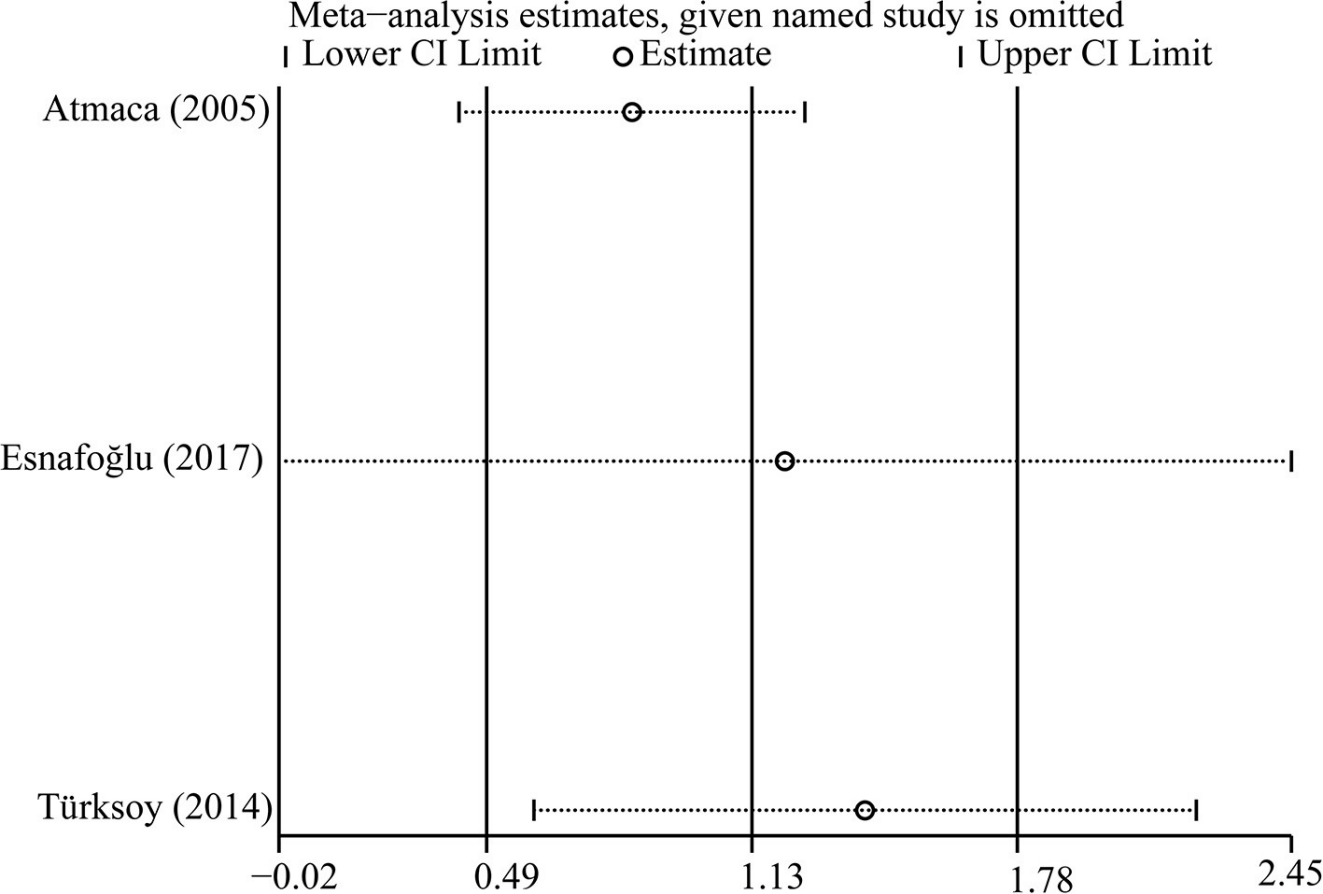
Sensitivity analysis of included studies on homocysteine.

**Figure 7:**
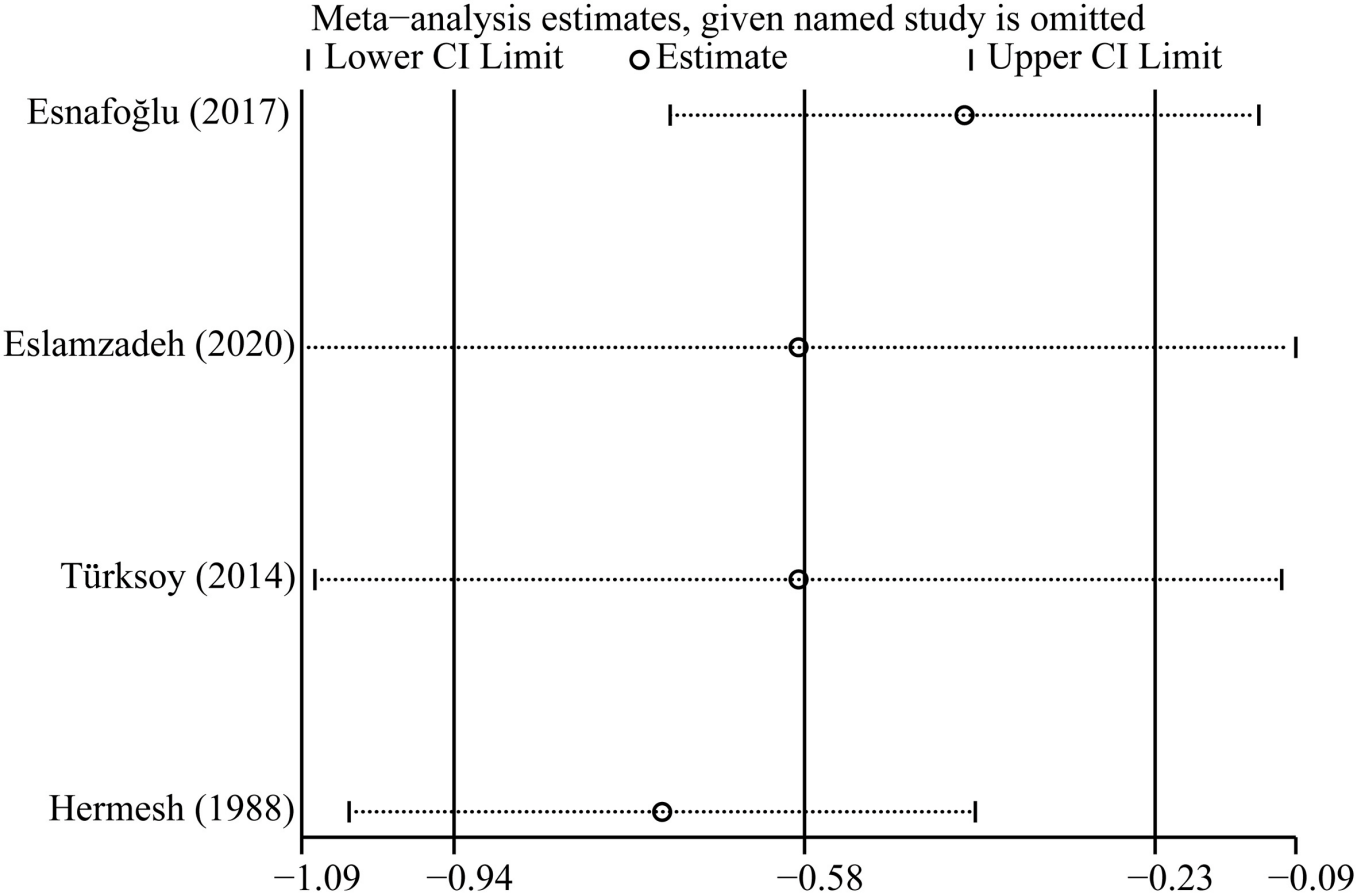
Sensitivity analysis of included studies on vitamin B12.

There was no obvious influence of one individual study on the pooled SMDs of serum levels of folate, vitamin B12, and homocysteine in groups (Figures 5–7).

## Discussion

Obsessive–compulsive disorder is a complex neuropsychiatric disorder with not fully elucidated etiology (6, 7, 11, 21). Studies have shown that anomalous changes in serum levels of vitamin B12, folate, and homocysteine may contribute to the development of OCD (37–40). In this meta-analysis, we found a statistically significant higher homocysteine level and lower concentration of B12 vitamins in patients with OCD. This result is in line with most of the findings (37–40). However, there was not a statistically significant correlation between folate deficiency and OCD. This is consistent with the results of the 3 studies (38, 40, 43) and contradicts the results of the other 2 studies (37, 39). Although there were differences in the content of serum levels of vitamin B12 and homocysteine between the patients with OCD and normal individuals, whether these potential influences could be proved in the future by well-designed RCTs is still unknown.

The synthesis of methionine from homocysteine requires a supply of methyl groups from methyl folate. Folate and vitamin B12 are required in the synthesis of SAM, the sole methyl donor in numerous methylation reactions involving proteins, phospholipids, and biogenic amines. In the case of folate or vitamin B deficiency, the S-adenosylmethionine synthesis may become severely impaired, which might be the cause of many neuropsychiatries (28, 44–46). Reduced synthesis of S-adenosylmethionine could be due to a hypo-methylation state, leading in turn to impaired synthesis of proteins and neurotransmitters necessary for the brain structural integrity, and OCD was further induced (47). Decreased folate levels will elevate homocysteine levels. In addition, the methyl producing is indirectly involved in nucleotide, DNA, and RNA synthesis and is critical for many genomic and non-genomic methylation reactions. Disruption of methylation (single carbon transfer) responses leads to the production of monoamine neurotransmitters, phospholipids, and nucleotides in the central nervous system, which might be a pathologic mechanism for depression. In addition, folate deficiency may help mediate some of its neuropsychiatric complications by generating elevated levels of Sadenosyl-homocysteine, which broadly inhibits methylation reactions, and possibly exert direct excitotoxic effects *via* activity at the N-methyl-D-aspartate glutamate receptors (48, 49). Furthermore, this hypomethylation state can lead to impaired synthesis of proteins and neurotransmitters required for the brain's structural integrity (47, 50).

Observations on the antidepressant effects of folate supplementation may suggest the effect of these nutrients in psychopathology (51, 52). Folate also considerably influences the rate of synthesis of tetrahydrobiopterin (53–55), a cofactor in the hydroxylation of phenylalanine and tryptophan, rate-limiting steps in the biosynthesis of dopamine, norepinephrine, and serotonin, neurotransmitters postulated to impact the monoamine hypothesis of affective disorders. In addition, there is a study supporting that genetic modifications affecting the MTFHR level or function decreases the 5-methyltetrahydrofolate level further results in increased homocysteine (49, 56, 57). There are many meta-analyses in the literature that reveal the correlation between psychiatric diseases and MTHFR gene polymorphisms (58–61). It is also a non-negligible reason for OCD. SAM^3^ and methyl folate have been shown to exert stronger antidepressant effects than placebos when administered in parenteral and certain oral forms, and are even more effective than tricyclic antidepressants (62–64). Total plasma homocysteine can be as a sensitive marker of functional deficiency of folate and vitamin B12 (65, 66). Total homocysteine level changes have also been suggested to be correlated with numerous psychiatric disorders (e.g., schizophrenia and affective disorders) (61, 67–72). Also, some literature reported neuropsychiatric symptoms in patients with vitamin B12 deficiency, including ataxia, mania, hallucinations, memory loss, depression, paresthesias, proprioception loss, delirium, dementia, personality change, and abnormal behavior (73–76). As mentioned earlier, folate and vitamin B12 deficiencies inhibit the methylation reactions, as well as elevate homocysteine levels, which results in a drop in neurotransmitter levels and affects other biochemical pathways in the cell to varying adversely degrees (77, 78).

This meta-analysis has the following limitations that should be considered: (1) some articles' measures data are not distributed normally and were not reported in the form of median and quartile, and therefore, they could not be included in the meta-analysis; (2) only English and Chinese language reports have been searched and maybe consequently leads to missing data from other vital high-quality articles published in other languages; (3) since all the included studies were retrospective with small sample sizes, high-quality RCTs with large sample is lacking; (4) the results were based on unadjusted estimates, more accurate outcomes would be achieved due to adjustments for other confounding factors (e.g., gender, age, body mass index, and lifestyle); (5) some factors had not been taken consider into study, e.g., renal function of participants, dietary habits changes correlated with symptoms in patients with OCD, reduced ultraviolet exposure required for vitamin D synthesis; (6) one set of trails from the last century has not mentioned the details about the diagnosis and detection methods (43); (7) most of the participants in 1 study were women (40); (8) there are also some differences in the laboratory examination methods for each index in each group due to time. Various factors aforementioned may contribute to the heterogeneity of the data. In the future, more standardized patients should be involved and the principle of randomization and double blindness should be strictly followed to achieve the higher experimental effect.

## Conclusions

In conclusion, this meta-analysis found that OCD might be correlated with a low level of vitamin B12 and high level of homocysteine, whereas the sample here was too small to conclude that this change might be a vital biological indicator for OCD. Abnormal serum levels of vitamin B12 and homocysteine expression might be a risk factor for the development of OCD. Clinicians should be vigilant about serum levels of vitamin B12 and homocysteine in patients with OCD, which should be investigated in more in-depth and high-quality studies. These findings can provide a starting point for further research.

## Data Availability Statement

The original contributions presented in the study are included in the article/supplementary materials, further inquiries can be directed to the corresponding author/s.

## Author Contributions

SY: conceptualization, methodology, article selection, data extraction, data analysis, quality assessment, and writing—original draft preparation. HL and YY: data extraction and quality assessment. NH: quality assessment. All authors contributed to the article and approved the submitted version.

## Conflict of Interest

The authors declare that the research was conducted in the absence of any commercial or financial relationships that could be construed as a potential conflict of interest.

## Publisher's Note

All claims expressed in this article are solely those of the authors and do not necessarily represent those of their affiliated organizations, or those of the publisher, the editors and the reviewers. Any product that may be evaluated in this article, or claim that may be made by its manufacturer, is not guaranteed or endorsed by the publisher.

## Footnotes

^1^OCD, obsessive-compulsive disorder.

^2^GABA, γ-aminobutyric acid.

^3^SAM, S-adenosylmethionine.
